# Caloric restriction extends yeast chronological lifespan via a mechanism linking cellular aging to cell cycle regulation, maintenance of a quiescent state, entry into a non-quiescent state and survival in the non-quiescent state

**DOI:** 10.18632/oncotarget.20614

**Published:** 2017-09-01

**Authors:** Anna Leonov, Rachel Feldman, Amanda Piano, Anthony Arlia-Ciommo, Vicky Lutchman, Masoumeh Ahmadi, Sarah Elsaser, Hana Fakim, Mahdi Heshmati-Moghaddam, Asimah Hussain, Sandra Orfali, Harshvardhan Rajen, Negar Roofigari-Esfahani, Leana Rosanelli, Vladimir I. Titorenko

**Affiliations:** ^1^ Department of Biology, Concordia University, Montreal, Quebec, Canada

**Keywords:** yeast, cellular aging, cell cycle, cell differentiation, cell quiescence, Gerotarget

## Abstract

A yeast culture grown in a nutrient-rich medium initially containing 2% glucose is not limited in calorie supply. When yeast cells cultured in this medium consume glucose, they undergo cell cycle arrest at a checkpoint in late G1 and differentiate into quiescent and non-quiescent cell populations. Studies of such differentiation have provided insights into mechanisms of yeast chronological aging under conditions of excessive calorie intake. Caloric restriction is an aging-delaying dietary intervention. Here, we assessed how caloric restriction influences the differentiation of chronologically aging yeast cultures into quiescent and non-quiescent cells, and how it affects their properties. We found that caloric restriction extends yeast chronological lifespan via a mechanism linking cellular aging to cell cycle regulation, maintenance of quiescence, entry into a non-quiescent state and survival in this state. Our findings suggest that caloric restriction delays yeast chronological aging by causing specific changes in the following: 1) a checkpoint in G1 for cell cycle arrest and entry into a quiescent state; 2) a growth phase in which high-density quiescent cells are committed to become low-density quiescent cells; 3) the differentiation of low-density quiescent cells into low-density non-quiescent cells; and 4) the conversion of high-density quiescent cells into high-density non-quiescent cells.

## INTRODUCTION

A body of knowledge about mechanisms underlying chronological aging of the yeast *Saccharomyces cerevisiae* has been provided by studies in which yeast cells were cultured in a nutrient-rich liquid medium initially containing 2% glucose [1, 2]. Under these so-called non-caloric restriction (non-CR) conditions yeast cells are not limited in the supply of calories [1, 3, 4]. When glucose is exhausted at the diauxic shift, cells in a non-CR yeast culture undergo arrest at the G_1_ phase of the cell cycle. The non-CR yeast culture then differentiates into several cell populations [5-8].

One of these cell populations is a population of quiescent (Q) cells; these cells exist in a distinct non-proliferative state called G_0_ [5-11]. Q cells are mainly daughter cells [5-7]. They are unbudded and uniformly sized, are refractive by phase-contrast microscopy and enclosed by a rigid cell wall, have high buoyant density, store glycogen and trehalose in bulk quantities, are highly metabolically active, exhibit high rates of mitochondrial respiration and low concentrations of reactive oxygen species (ROS), are able to form colonies when plated on fresh solid medium, can re-enter mitosis when nutrients become available following transfer to fresh liquid medium, are resistant to long-term thermal and oxidative stresses, exhibit low rates of mutations that impair mitochondrial functionality, and display a delayed onset of the apoptotic and necrotic modes of programmed cell death (PCD) [5-8, 10, 11].

The differentiation of a non-CR yeast culture following glucose exhaustion at the diauxic shift also yields at least three subpopulations of non-quiescent (NQ) cells, most or all of which are first- and higher-generation mother cells [5-8, 10, 11]. One subpopulation of NQ cells consists of metabolically active cells that exhibit high reproductive (colony-forming) capacities, high ROS concentrations, impaired mitochondrial respiration and elevated frequencies of mutations impairing mitochondrial functionality [5-8, 10, 11]. Another subpopulation of NQ cells includes metabolically active cells that are impaired in reproductive (clonogenic) ability and are likely to be descended from NQ cells of the first subpopulation [5-8, 10, 11]. The third subpopulation of NQ cells is composed of cells that exhibit hallmarks of the apoptotic and/or necrotic modes of PCD and may derive from NQ cells of the second subpopulation [5-8, 10, 11].

In response to a depletion of glucose (as well as nitrogen, phosphate or sulfur), a signaling network of certain proteins and protein complexes orchestrates cell cycle arrest at the G_1_ phase of the cell cycle, the differentiation of a chronologically aging non-CR yeast culture into populations of Q and NQ cells, and quiescence maintenance. Proteins and protein complexes integrated into this signaling network operate as network nodes, many of which are connected by physical links known to be predominantly phosphorylations and dephosphorylations that activate or inhibit specific target proteins [9, 12-17]. The core hubs of this signaling network of a quiescence program are four nutrient-sensing protein complexes, each of which exhibits a protein kinase activity and modulates many downstream effector proteins integrated into the network. These core hubs of the network are: 1) TORC1 (target of rapamycin complex 1), a key regulator of cell metabolism, growth, division and stress resistance in response to changes in the availabilities of nitrogen and carbon sources; 2) PKA (protein kinase A), an essential controller of cell metabolism, proliferation and stress resistance in response to changes in carbon source availability; 3) Snf1 (sucrose non-fermenting, protein 1), a heterotrimeric protein complex required for cell growth support and energy homeostasis maintenance after glucose exhaustion; and 4) Pho85 (phosphate metabolism, protein 85), a protein kinase associated with various cyclins to promote phosphate metabolism, glycogen and trehalose synthesis, oxidative stress response and cellular proteostasis in response to changes in the accessibility of a phosphate source or following glucose exhaustion [9, 12, 14, 18, 19].

The four core hubs of the signaling network of the quiescence program modulate many downstream effector proteins, including the following: 1) Rim15, a serine-threonine protein kinase which, following glucose exhaustion at the diauxic shift, is essential for cell cycle arrest at G_1_, cell survival during stationary growth phase, transcription of many stress response genes, trehalose and glycogen accumulation, autophagy, and post-transcriptional protection of a subset of mRNAs needed for entry into quiescence; Rim15 is controlled by the TORC1, PKA and Pho85 core hubs of the network [18, 20-31]; 2) Sch9, a serine-threonine protein kinase which, prior to glucose exhaustion at the diauxic shift, prevents entry into quiescence by stimulating transcription of genes essential for ribosome biogenesis, promoting translation initiation and suppressing transcription of many stress response genes; Sch9 is modulated by TORC1 [16, 18, 23, 28, 32-36]; 3) Yak1, a serine-threonine protein kinase which in yeast cells entering SP phase is required for cell cycle arrest at G_1_, suppresses transcription of genes encoding ribosomal proteins and activates transcription of many stress response genes; Yak1 is under the control of PKA [37-41]; 4) Mck1, a dual-specificity serine-threonine and tyrosine protein kinase which, after glucose exhaustion at the diauxic shift, is essential for the accumulation of trehalose and glycogen, ROS detoxification and transcription of many stress response genes; Mck1 is likely to be controlled by TORC1 and PKA [42-44]; 5) Msn2/4 and Gis1, transcription factors that activate expression of stress-responsive element- and post-diauxic shift-controlled (respectively) genes involved in protection against thermal, oxidative and osmotic stresses, as well as in carbohydrate metabolism; these transcription factors are modulated by TORC1, PKA and Snf1 [25, 26, 29, 45-55]; 6) Hsf1, a transcription factor which activates expression of many heat shock element-controlled genes involved in protein folding, protein synthesis and modification, ROS detoxification, energy generation, carbohydrate metabolism, intracellular vesicular trafficking, and cell wall maintenance; Hsf1 is controlled by Snf1 [56-59]; 7) Gln3, a transcriptional activator of nitrogen catabolite-repressible genes involved in the metabolism and transport of alternative nitrogen sources; Gln3 is under the control of TORC1 and Snf1 [47, 60-64]; 8) Gsy2, glycogen synthase which is induced upon glucose exhaustion or nitrogen starvation; Gsy2 is modulated by TORC1, PKA, Snf1 and Pho85 [63, 65-72]; 9) the Atg1-Atg13 complex, which initiates autophagy by enabling phagophore assembly site formation; this complex is controlled by TORC1, PKA, Snf1 and Pho85 [73-80]; 10) Sfp1, a transcription activator of genes encoding ribosomal proteins and protein components of ribosome biogenesis machinery; Sfp1 is under the control of TORC1 and PKA [33, 35, 76, 81-87]; 11) eIF2α, a subunit of a protein complex involved in the initiation of protein synthesis on the ribosome; eIF2α is modulated by TORC1 and Snf1 [88, 89]; ]; 12) Crz1, a transcription factor activating expression of stress response genes; Crz1 is modulated by PKA and Pho85 [90-94]; 13) Igo1 and Igo2, two paralogues proteins whose Rim15-diven phosphorylation upon glucose exhaustion protects from degradation a specific set of mRNAs required for entry into quiescence; phosphorylated Igo1 and Igo2 also inhibit the Cdc55 protein phosphatase 2A (PP2A^Cdc55^) to prevent dephosphorylation of the transcription factor Gis1, thereby enabling phosphorylated Gis1 to activate transcription of many stress response and carbohydrate metabolism genes, as well as transcription of the *XBP1* gene encoding a global transcriptional repressor of genes involved in cell cycle progression, transition to the Q state and recovery from it; additionally, the Igo1/2-dependent inhibition of PP2A^Cdc55^ prevents dephosphorylation of the cyclin-dependent kinase (CDK) inhibitor Sic1 to protect it from proteolysis - so that phosphorylated Sic1 can elicit cell cycle arrest at late G_1_ [8, 30, 31, 95-101]; and 14) Mpk1, a mitogen-activated protein kinase which phosphorylates the CDK inhibitor Sic1 to prevent its proteolytic degradation and to allow phosphorylated Sic1 to assist in arresting cell cycle at a checkpoint in late G_1_; Mpk1 is controlled by TORC1 [100, 101].

CR is a dietary intervention that delays aging in yeast and other evolutionarily distant eukaryotic organisms [1, 2, 102-106]. Although mechanisms linking yeast chronological aging to the quiescence program under non-CR conditions are well established [5-9, 11, 43, 44, 97, 107], it is unknown if the aging-delaying effect of CR in chronologically aging yeast is due in part to the ability of this low-calorie diet to control the quiescence program. In this study, we provide evidence that CR slows down yeast chronological aging through a mechanism which affects several key aspects of the quiescence program.

## RESULTS

### CR accelerates an age-related accumulation of low-density cells in chronologically aging yeast cultures

**Figure 1:**
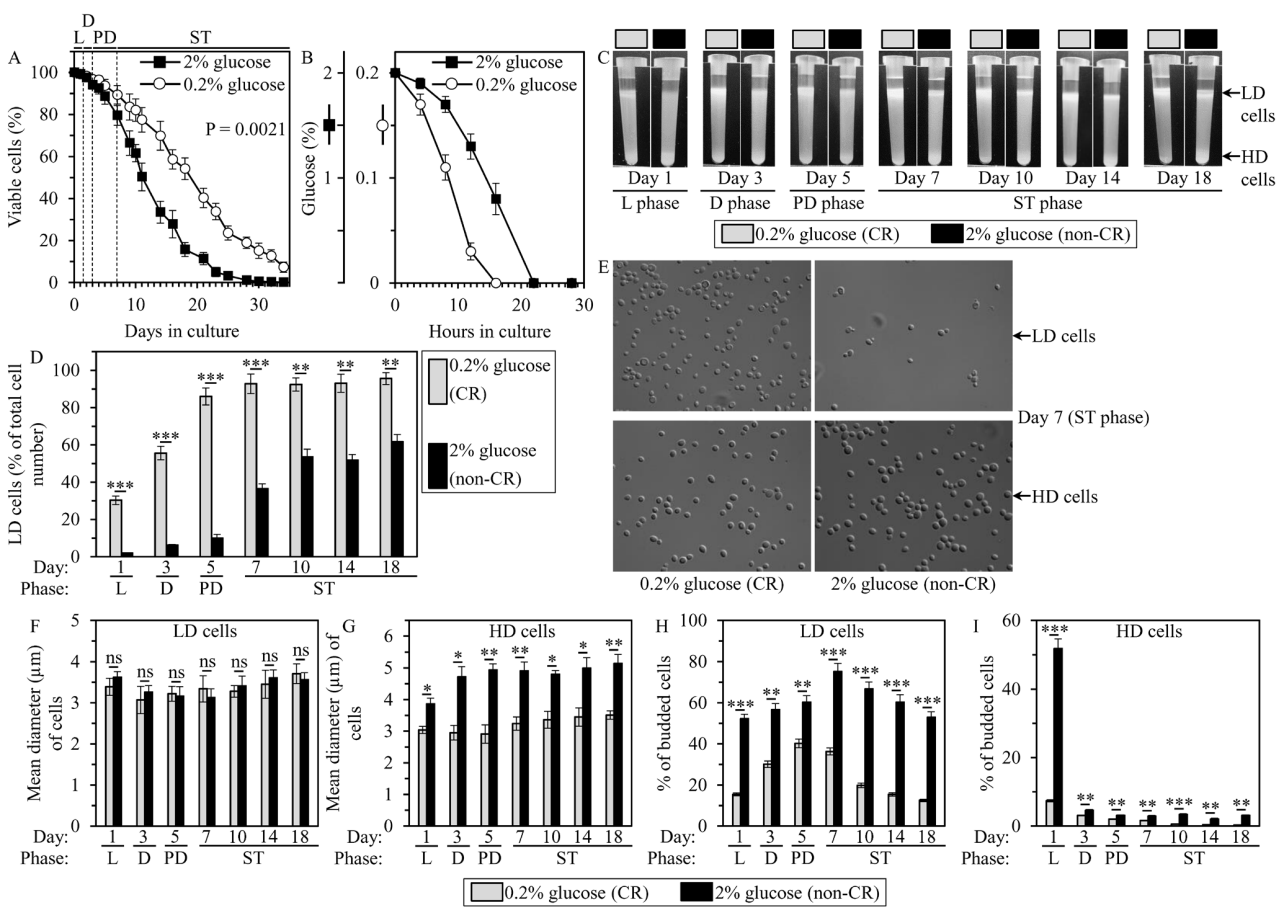
Caloric restriction (CR) accelerates an age-related accumulation of low-density (LD) cells, decreases the size of high-density (HD) cells, and lowers the abundance of budded cells in LD and HD populations of chronologically aging yeast cultures **A.** Survival of chronologically aging wild-type (WT) yeast cultured in the nutrient-rich YP medium initially containing 0.2% glucose (CR conditions) or 2% glucose (non-CR conditions). The logrank test for comparing each pair of survival curves was performed as described in Materials and Methods. Two survival curves were considered statistically different if the P value was less than 0.05. **B.** Kinetics of glucose consumption for WT yeast cultured in YP medium initially containing 0.2% glucose (CR conditions) or 2% glucose (non-CR conditions). **C.** to **I.** Samples of WT yeast cultured in YP medium initially containing 0.2% glucose (CR conditions) or 2% glucose (non-CR conditions) were recovered from logarithmic (L), diauxic (D), post-diauxic (PD) or stationary (ST) growth phase and subjected to centrifugation in Percoll density gradient as described in Materials and Methods. Percoll density gradients (C), the percentage of LD cells (D), differential interference contrast micrographs of LD and HD cells recovered from ST growth phase on day 7 (E), mean diameters of LD (F) or HD (G) cells, and the percentages of budded cells present in LD (H) or HD (I) populations are shown. Data in A, B, D, F - I are presented as means ± SEM (*n* = 3; *P < 0.05; **P < 0.01; ***P < 0.001; ns, not significant).

Chronologically aging yeast cultured under CR conditions in the nutrient-rich YP medium initially containing 0.2% glucose lived significantly longer than yeast undergoing chronological aging under non-CR conditions in YP medium initially supplemented with 2% glucose (Figure 1A). Even before glucose is depleted from the medium at the diauxic shift, a yeast culture undergoing chronological aging under non-CR conditions is known to differentiate into cell populations with different buoyant densities [6, 8]. Each of these cell populations can be purified to homogeneity by centrifugation in Percoll density gradient [5].

Using centrifugation in Percoll density gradient for separating cell populations exhibiting different densities, we investigated how CR influences the differentiation of a chronologically aging yeast culture into these distinct cell populations. Samples of cells cultured in YP medium initially containing 0.2% glucose (CR conditions) or 2% glucose (non-CR conditions) were recovered at different time-points following glucose exhaustion from the medium, which occurs 16 or 22 h after cell inoculation, respectively (Figure 1B). We found that chronologically aging cultures of CR and non-CR yeast 1) contain both low-density (LD) and high-density (HD) cell populations during logarithmic (L), diauxic (D), post-diauxic (PD) and stationary (ST) growth phases, i.e. through the entire chronological lifespan; and 2) exhibit an age-related increase in the percentage of LD cells (Figures 1C and 1D). We also noticed that the percentage of LD cells in CR yeast cultures significantly exceeds that in non-CR yeast cultures during any of the four growth phases, i.e. at any stage of the chronological aging process (Figure 1C and 1D). Moreover, we showed that the percentage of LD cells in CR yeast cultures reaches a plateau on day 5 of culturing (in PD growth phase), whereas the percentage of LD cells in non-CR yeast cultures attains a steady-state level only on day 10 of culturing (in ST growth phase) (Figure 1C and 1D).

Collectively, these findings indicate that CR accelerates an age-related accumulation of LD cell population in chronologically aging yeast cultures.

### CR alters cell size and the abundance of budded cells in LD and HD populations

We then assessed how CR influences the morphology of cells present in LD and HD populations. These cell populations were first recovered from yeast cultures of different chronological ages and then separated from each other by centrifugation in Percoll density gradient. Our differential interference contrast (DIC) microscopical examination and subsequent morphometric analysis of these LD and HD cell populations have revealed that through the entire chronological lifespan: 1) LD cells in CR cultures have sizes similar to those of LD cells in non-CR cultures (Figure 1E and 1F; Supplementary Figure 1); 2) HD cells in CR cultures remain significantly smaller than HD cells in non-CR cultures (Figure 1E and 1G; Supplementary Figure 1); and 3) although LD cells in CR and non-CR cultures contain both budded and unbudded cells, the percentage of budded LD cells in CR cultures is significantly lower than that in non-CR cultures (Figure1E and 1H; Supplementary Figure 1). We also found that, with the exception of HD cells recovered from non-CR cultures in L growth phase, most of HD cells recovered from CR and non-CR cultures were unbudded (Figure 1E and 1I; Supplementary Figure 1). Moreover, we showed that the percentage of budded HD cells in CR cultures is significantly lower than that in non-CR cultures (Figure 1E and 1I; Supplementary Figure 1).

In sum, these findings indicate that CR decreases the size of HD cells and lowers the percentage of budded cells in both LD and HD populations through the entire process of chronological aging.

### CR delays an age-related decline in the reproductive proficiencies of LD and HD cell populations and in their abilities to synchronously re-enter mitosis

Our observation that nearly all HD cells in CR and non-CR cultures were unbudded (except of HD cells purified from non-CR cultures in L growth phase, see Figure 1I) suggests a hypothesis that HD population contains predominantly Q cells. Q cells are known to be mainly unbudded [5]. Moreover, because LD population recovered from CR and non-CR cultures contained both budded and unbudded cells (Figure 1H), we hypothesized that LD population consists mainly of NQ cells. NQ cells are known to represent a mixture of budded and unbudded cells [5].

To test this hypothesis, we compared the following two features of LD and HD populations: 1) the reproductive competence, i.e. the ability of a yeast cell to form a colony when plated on fresh solid medium; and 2) the ability of a yeast cell population to synchronously re-enter the mitotic cell cycle if returned to growth-promoting conditions. Both these abilities are known to be characteristic of Q cells; these fundamental characteristics of Q cells distinguish them from NQ cells [7]. We recovered LD and HD cell populations from CR or non-CR yeast cultures of different chronological ages and then separated them from each other by centrifugation in Percoll density gradient.

**Figure 2:**
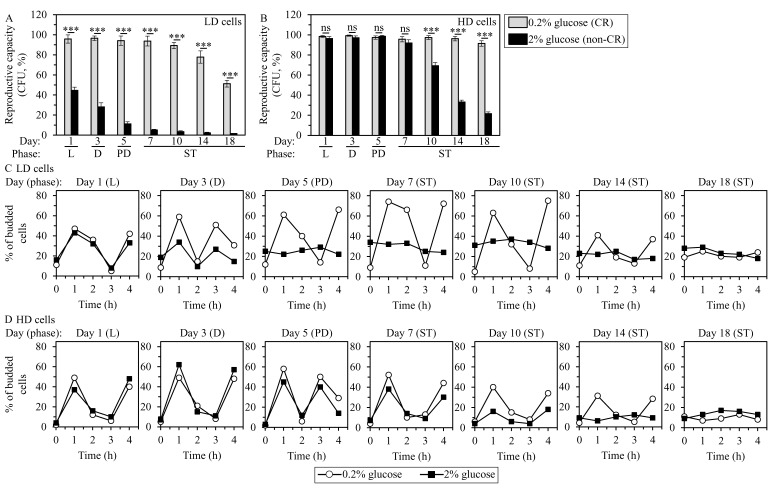
CR delays an age-related decline of the reproductive competences of LD and HD cells and of their capabilities to synchronously re-enter the mitotic cell cycle when nutrients become available **A.** to **D.** Samples of WT yeast cultured in YP medium initially containing 0.2% glucose (CR conditions) or 2% glucose (non-CR conditions) were recovered from L, D, PD or ST growth phase and subjected to centrifugation in Percoll density gradient to purify LD and HD cell populations, as described in Materials and Methods. The reproductive (colony forming) efficiencies of LD (A) or HD (B) cells, and the efficiencies with which LD (C) or HD (D) cells can synchronously re-enter the mitotic cell cycle after cell transfer into fresh medium and incubation for 1 to 4 h are shown. These efficiencies were measured as described in Materials and Methods. Data in A and B are presented as means ± SEM (*n* = 3; ***P < 0.001; ns, not significant). Data in C and D are presented as means (*n* = 3 - 4).

Our comparative analyses of the reproductive (colony-forming) capacities of LD and HD cells purified from differently aged CR and non-CR yeast cultures have revealed the following: 1) LD cells in CR cultures maintain reproductive (colony-forming) capacity for a long period of time in the course of chronological aging, whereas LD cells in non-CR cultures exhibit a very rapid age-related deterioration in reproductive competence (Figure 2A), and 2) HD cells in CR cultures sustain reproductive (colony-forming) ability through the entire process of chronological aging, whereas HD cells in non-CR cultures display an age-related gradual decline in reproductive capability (Figure 2B).

Our comparison of the abilities of LD and HD cells purified from differently aged CR and non-CR yeast cultures to synchronously re-enter the mitotic cell cycle after cell transfer into fresh medium and incubation for 1 to 4 h has revealed the following: 1) LD cells in CR cultures remain Q cells (i.e. maintain the ability to synchronously re-enter mitosis following cell transfer) for a long period of time in the course of chronological aging, whereas LD cells in non-CR cultures become NQ cells (i.e. senescent) soon after being formed in the process of differentiation (Figure 2C); and 2) HD cells in CR cultures continue to be Q cells through most of the chronological aging process, whereas HD cells in non-CR cultures progressively become NQ cells (Figure 2D).

Taken together, these findings provide evidence that 1) HD population contains predominantly Q cells, whereas LD population consists mainly of NQ cells; and 2) CR slows an age-related deterioration in the reproductive competences of Q and NQ cells and in their capabilities to synchronously re-enter the mitotic cell cycle when nutrients become available.

### CR increases the abundance of daughter cells in Q and NQ populations

A culture of the budding yeast *S. cerevisiae* is known to contain first- and higher-generation daughter cells without bud scars and first- and higher-generation mother cells with one or more bud scars on the cell surface [108-110]. Each bud scar marks a division site on the surface of a mother cell and, thus, the number of bud scars accumulating on the mother cell surface is a measure of its replicative age [111-113].

**Figure 3:**
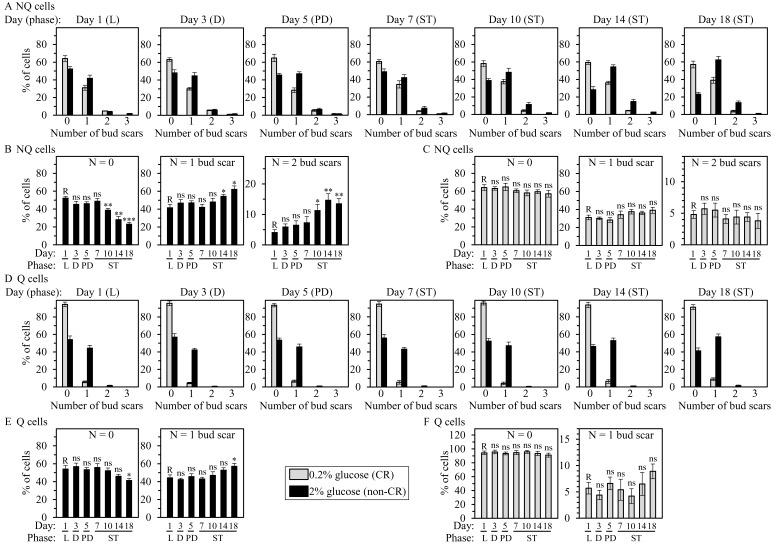
CR increases the abundance of daughters in Q and NQ cell populations through the entire chronological lifespan and prevents budding of daughters in these cell populations late in life **A.** to **F.** Samples of WT yeast cultured in YP medium initially containing 0.2% glucose (CR conditions) or 2% glucose (non-CR conditions) were recovered from L, D, PD or ST growth phase and subjected to centrifugation in Percoll density gradient to purify Q and NQ cell populations, as described in Materials and Methods. Bud scars were microscopically visualized by staining with Calcofluor White M2R, as described in Materials and Methods. The percentages of cells with 0, 1, 2 or 3 bud scars in NQ (A to C) or Q (D to F) populations of different chronological ages are shown. Data in B, C, E and F are presented as means ± SEM (*n* = 3; *P < 0.05; **P < 0.01; ***P < 0.001; ns, not significant; R, reference). Data in A and D are presented as means (*n* = 3 - 5).

We assessed the relative abundance of first- and higher-generation daughters and replicatively aged mothers present in Q and NQ cell populations. These cell populations were first recovered from CR or non-CR yeast cultures of different chronological ages and then separated from each other by centrifugation in Percoll density gradient. Bud scars were microscopically visualized by staining with Calcofluor White M2R. Our comparative analysis of Q and NQ cell populations purified from differently aged CR and non-CR cultures has revealed the following: 1) CR increases the abundance of first- and higher-generation daughters and decreases the percentage of first- and higher-generation mothers present in NQ cell populations through the entire chronological lifespan (Figure 3A; Supplementary Figure 2); 2) the abundance of daughter cells decreases and the abundance of mother cells increases in NQ cell populations from non-CR cultures recovered on days 10, 14 and 18 of culturing (Figure 3B; Supplementary Figure 2); 3) the percentages of daughter and mother cells remain unchanged through the entire chronological lifespan in NQ cell populations from CR cultures (Figure 3C; Supplementary Figure 2); 4) through the entire chronological lifespan, Q cell population in CR cultures is predominantly composed of daughter cells whereas Q cell population in non-CR cultures is a mixture of daughter and mother cells (Figure 3D; Supplementary Figure 2); 5) the abundance of daughter cells decreases and the abundance of mother cells increases in the Q cell population that was purified from non-CR cultures recovered very late in chronological lifespan, namely on day 18 of culturing (Figure 3E; Supplementary Figure 2); and 6) the percentages of daughter and mother cells remain unaltered through the entire chronological lifespan in Q cell populations purified from CR cultures (Figure 3F; Supplementary Figure 2).

Collectively, these findings indicate that 1) through the entire chronological lifespan, CR rises the fraction of daughter cells in Q and NQ populations; and 2) late in chronological lifespan, CR prevents budding of daughter cells in NQ population and, to a lesser extent, in Q population.

### CR increases the concentrations of glycogen and trehalose in Q and NQ cell populations

One of the metabolic hallmarks of Q cells that distinguish them from NQ cells is an accumulation of glycogen and trehalose, the two major glucose stores in yeast [5, 114]. In addition to being reserve carbohydrate, trehalose has also been implicated in protecting yeast cells and cellular proteins from oxidative and other stresses [115-118], modulating cellular proteostasis [119-121], and allowing Q cells to re-enter the mitotic cell cycle when nutrients become available [114].

**Figure 4:**
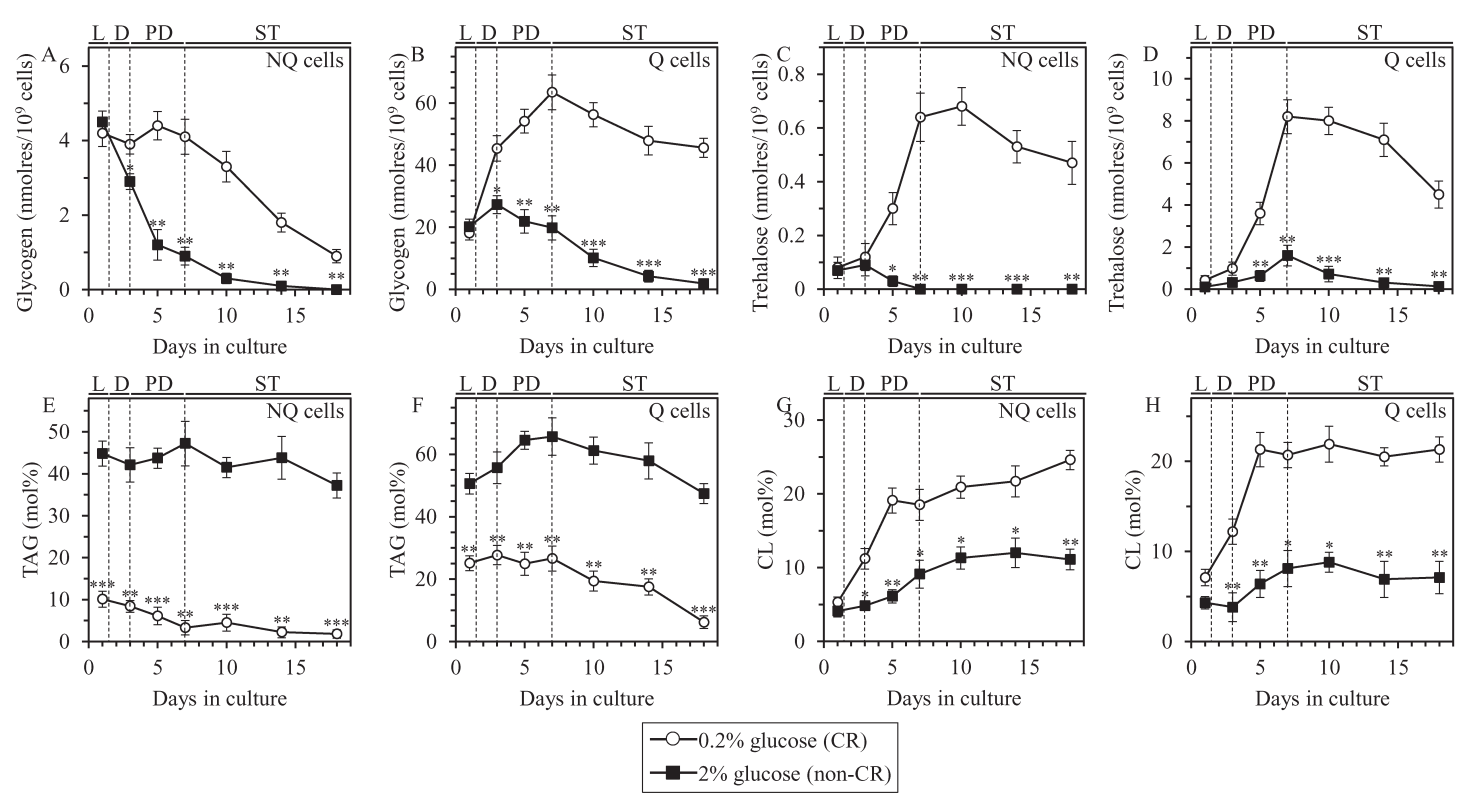
CR alters the abundance of glycogen, trehalose, triacylglycerols (TAG) and cardiolipins (CL) in Q and NQ cell populations through most of the chronological lifespan Samples of WT yeast cultured in YP medium initially containing 0.2% glucose (CR conditions) or 2% glucose (non-CR conditions) were recovered from L, D, PD or ST growth phase and subjected to centrifugation in Percoll density gradient to purify Q and NQ cell populations, as described in Materials and Methods. The concentrations of glycogen **A.** and **B.**, trehalose **C.** and **D.**, TAG **E.** and **F.** and CL **G.** and **H.** were measured as described in Materials and Methods. Data are presented as means ± SEM (*n* = 3; *P < 0.05; **P < 0.01; ***P < 0.001).

We investigated how CR influences the intracellular concentrations of glycogen and trehalose in Q and NQ cell populations. These cell populations were first recovered from CR or non-CR yeast cultures of different chronological ages and then separated from each other by centrifugation in Percoll density gradient. Our comparative analysis of Q and NQ cell populations purified from differently aged CR and non-CR cultures has revealed the following: 1) through most of the chronological lifespan (i.e. since day 3 of culturing), the concentrations of glycogen in NQ and Q cells from CR cultures significantly exceed those in NQ and Q cells from age-matched non-CR cultures (Figure 4A and 4B, respectively); 2) through the entire chronological lifespan, the concentrations of glycogen in Q cells from CR cultures were higher than those in NQ cells from CR cultures of the same chronological age (compare Figure 4A and 4B); 3) for the most part (i.e. since day 5 of culturing), the concentrations of trehalose in NQ and Q cells from CR cultures considerably surpass those in NQ and Q cells from age-matched non-CR cultures (Figure 4C and 4D, respectively); and 4) through the entire chronological lifespan, the concentrations of trehalose in Q cells from CR cultures exceed those in NQ cells from CR cultures of the same chronological age (compare Figure 4C and 4D).

In sum, these findings indicate that through most of the chronological aging process CR elicits significant rises in the concentrations of glycogen and trehalose in both Q and NQ cell populations.

### CR alters the abundance of two lipid classes in Q and NQ cell populations

Recent studies have revealed that the longevity-extending effect of CR in chronologically aging yeast depends on the cellular homeostasis of two classes of lipids, namely triacylglycerols (TAG) and cardiolipins (CL) [2, 4, 122-135]. TAG are so-called neutral lipids that in yeast are synthesized in the endoplasmic reticulum and then deposited in lipid droplets to serve as the main storage molecules for maintaining energy homeostasis and supplying free fatty acids [136-139], whereas CL are signature lipids of the inner mitochondrial membrane implicated in oxidative phosphorylation and several other vital processes confined to mitochondria [140-143].

We examined the effect of CR on the abundance of TAG and CL in Q and NQ cell populations that were first recovered from CR or non-CR yeast cultures of different chronological ages and then purified by centrifugation in Percoll density gradient. Our comparison of these Q and NQ cell populations has revealed the following: 1) through the entire chronological lifespan, the concentrations of TAG in NQ and Q cells from CR cultures are significantly lower than those in NQ and Q cells from age-matched non-CR cultures (Figure 4E and 4F, respectively); 2) for the most part (i.e. since day 3 of culturing), the concentrations of CL in NQ and Q cells from CR cultures significantly exceed those in NQ and Q cells from non-CR cultures of the same chronological age (Figure 4G and 4H, respectively); 3) through the entire chronological lifespan, the concentrations of TAG in Q and NQ cells from age-matched CR and non-CR cultures are similar (compare Figure 4E and 4F); and 4) for the most part (i.e. since day 3 of culturing), the concentrations of CL in Q and NQ cells from CR and non-CR cultures of the same chronological age are also similar (compare Figure 4G and 4H).

We therefore concluded that through the entire chronological aging process CR induces a significant decline in the concentrations of TAG in Q and NQ cell populations. In contrast, through most of the chronological lifespan CR elicits a substantial rise in the abundance of CL in both these cell populations.

### CR alters the dynamics of age-related changes in mitochondrial functionality in Q and NQ cells

Mitochondrial electron transport chain, electrochemical potential across the inner mitochondrial membrane (ΔΨ_m_) and mitochondrial ROS play essential roles in defining longevity of chronologically aging yeast [1, 2, 4, 125, 135, 144-153]. Q cells purified from non-CR yeast cultures have been shown to exhibit high rates of mitochondrial respiration and low ROS, whereas NQ cells present in these cultures are known to have low rates of mitochondrial respiration and high ROS [5-7, 154].

We therefore investigated the effect of CR on some longevity-defining traits of mitochondrial functionality in Q and NQ cell populations recovered from CR or non-CR yeast cultures of different chronological ages and purified by centrifugation in Percoll density gradient. These traits included the rate of mitochondrial respiration, ΔΨ_m_ and cellular ROS, which in yeast are known to be produced primarily as by-products of mitochondrial respiration [155, 156].

**Figure 5:**
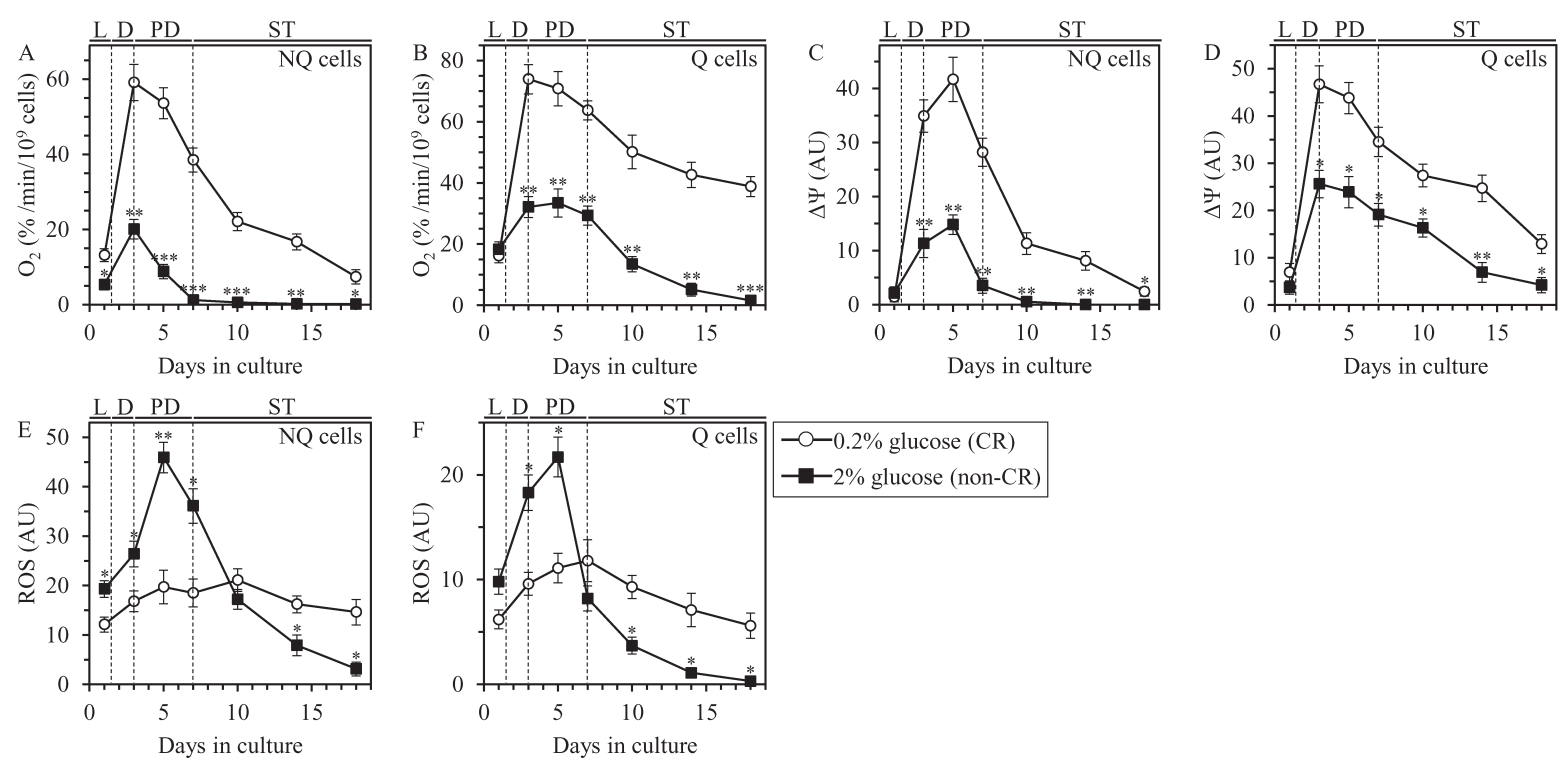
CR alters the patterns of age-related changes in certain traits of mitochondrial functionality in Q and NQ cells Samples of WT yeast cultured in YP medium initially containing 0.2% glucose (CR conditions) or 2% glucose (non-CR conditions) were recovered from L, D, PD or ST growth phase and subjected to centrifugation in Percoll density gradient to purify Q and NQ cell populations, as described in Materials and Methods. The rate of mitochondrial respiration **A.** and **B.**, ΔΨ_m_ **C.** and **D.** and ROS **E.** and **F.** were measured as described in Materials and Methods. Data are presented as means ± SEM (*n* = 3; *P < 0.05; **P < 0.01; ***P < 0.001).

Our comparative analyses of these traits of mitochondrial functionality in Q and NQ cells purified from differently aged CR and non-CR yeast cultures have revealed the following: 1) through the entire chronological lifespan, the rate of mitochondrial respiration in NQ cells from CR cultures is significantly higher than that in NQ cells from age-matched non-CR cultures (Figure 5A); 2) for the most part (i.e. since day 3 of culturing), the rate of mitochondrial respiration in Q cells from CR cultures substantially exceeds that in Q cells from age-matched non-CR cultures (Figure 5B); 3) through most of the chronological lifespan (i.e. since day 3 of culturing), the rates of mitochondrial respiration in NQ and Q cells from CR cultures are considerably higher than those in NQ and Q cells from non-CR cultures of the same chronological age (Figure 5C and 5D, respectively); 4) early in chronological lifespan (i.e. until day 7 for NQ cells or day 5 for Q cells) the concentrations of ROS in NQ and Q cells from CR cultures are notably lower than those in NQ and Q cells from age-matched non-CR cultures (Figure 5E and 5F, respectively); and 5) late in chronological lifespan (i.e. after day 10 for NQ cells or day 7 for Q cells) the concentrations of ROS in NQ and Q cells from CR cultures considerably exceed those in NQ and Q cells from non-CR cultures of the same chronological age (Figure 5E and 5F, respectively).

Taken together, these findings show that CR creates distinct patterns of mitochondrial functionality in Q and NQ cells by altering the chronology of age-related changes in mitochondrial respiration, ΔΨ_m_ and ROS.

### CR decreases the extent of age-related oxidative damage to proteins, lipids and DNA in Q and NQ cells

An age-related buildup of ROS-inflicted oxidative damage to various cellular macromolecules impairs their stability and is one of the major causes of aging in yeast and other eukaryotes [125, 157-164]. Q cells purified from non-CR yeast cultures are known to be genomically stable, whereas nuclear and mitochondrial DNA (nDNA and mtDNA, respectively) in NQ cells are likely instable because these cells exhibit an age-related excessive production of petite colonies [5, 7, 10]. Moreover, Q cells purified from non-CR yeast cultures do not accumulate SDS-insoluble protein aggregates, whereas NQ cells present in these cultures have been shown to amass such aggregates of irreversibly denatured/damaged proteins [106].

**Figure 6:**
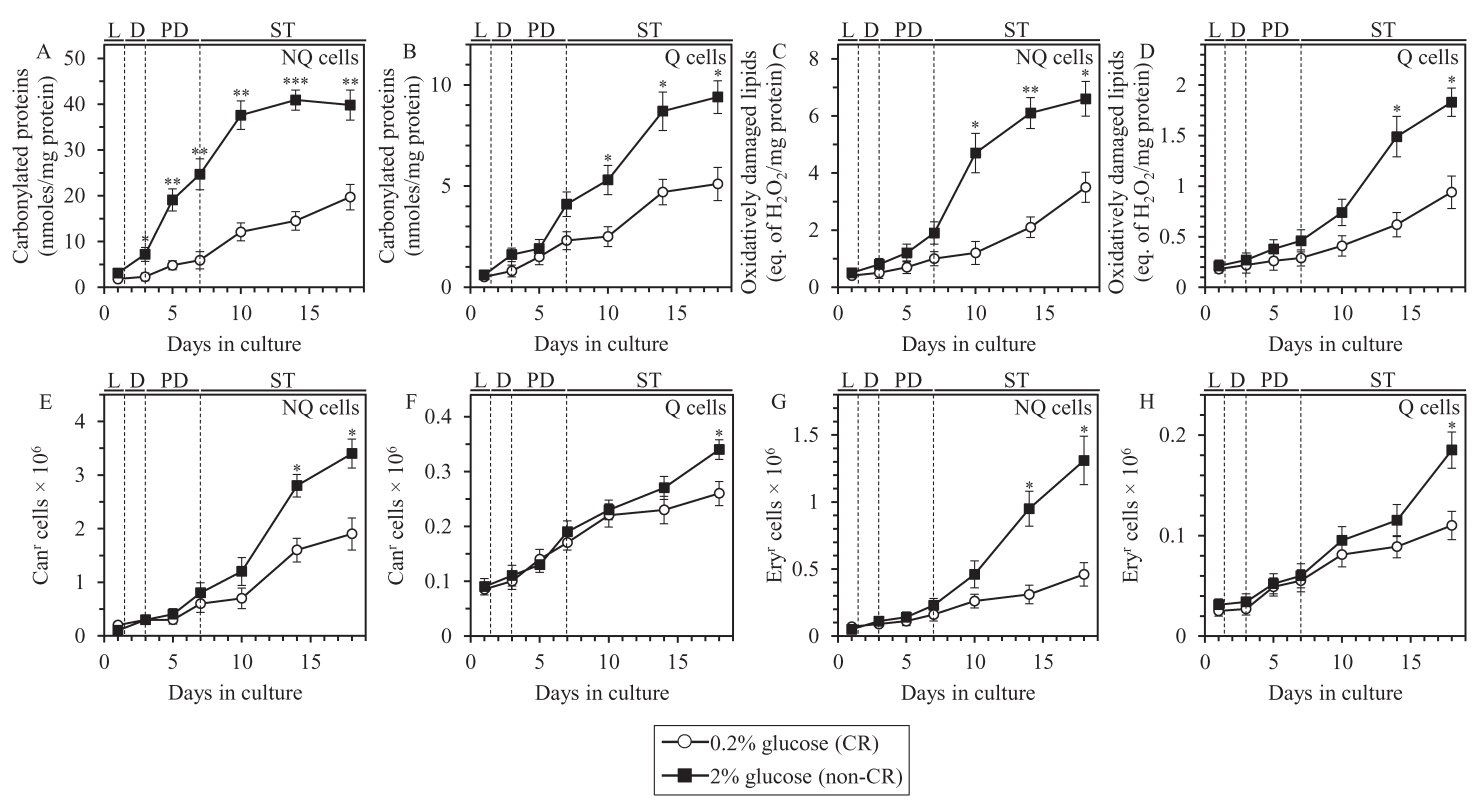
CR lessens the extent of age-related oxidative damage to proteins, lipids, nDNA and mtDNA in Q and NQ cells Samples of WT yeast cultured in YP medium initially containing 0.2% glucose (CR conditions) or 2% glucose (non-CR conditions) were recovered from L, D, PD or ST growth phase and subjected to centrifugation in Percoll density gradient to purify Q and NQ cell populations, as described in Materials and Methods. Carbonylated cellular proteins **A.** and **B.**, oxidatively damaged membrane lipids **C.** and **D.**, the frequencies of spontaneous point mutations in the *CAN1* gene of nDNA **E.** and **F**, and the frequencies of spontaneous point mutations in the *RIB2* and *RIB3* genes of mtDNA (G and H) were measured as described in Materials and Methods. Data are presented as means ± SEM (*n* = 3; *P < 0.05; **P < 0.01; ***P < 0.001).

We therefore analyzed how CR influences the extent of age-related oxidative damage to cellular proteins, membrane lipids, nDNA and mtDNA in Q and NQ cell populations that were recovered from differently aged CR or non-CR yeast cultures and purified by centrifugation in Percoll density gradient. Our analyses have revealed the following: 1) through most of the chronological lifespan (i.e. since day 3 of culturing), the extent of oxidative damage to proteins in NQ cells from CR cultures is considerably lower than that in NQ cells from age-matched non-CR cultures (Figure 6A); 2) late in chronological lifespan (i.e. since day 10) proteins in Q cells from CR cultures are oxidatively damaged to a lesser degree than proteins in Q cells from non-CR cultures of the same chronological age (Figure 6B); 3) late in chronological lifespan (i.e. after day 7 for NQ cells or after day 10 for Q cells), the extent of oxidative damage to membrane lipids in Q and NQ cells from CR cultures is significantly decreased as compared to that in NQ cells from age-matched non-CR cultures (Figure 6C and 6D, respectfully); 4) late in chronological lifespan (i.e. after day 10 for NQ cells or on day 18 for Q cells), the frequencies of spontaneous point mutations in the *CAN1* gene of nDNA in Q and NQ cells from CR cultures are substantially lower than those in Q and NQ cells from age-matched non-CR cultures - probably due to a decreased degree of oxidative damage to nDNA in Q and NQ cells from CR cultures (Figure 6E and 6F); and 5) late in chronological lifespan (i.e. after day 10 for NQ cells or on day 18 for Q cells), the frequencies of spontaneous point mutations in the *RIB2* and *RIB3* genes of mtDNA in Q and NQ cells from CR cultures are markedly decreased as compared to those in Q and NQ cells from age-matched non-CR cultures - perhaps due to a decline in the extent of oxidative damage to mtDNA in Q and NQ cells from CR cultures (Figure 6G and 6H).

In sum, these data imply that CR lessens the degree of age-related oxidative damage to proteins, lipids, nDNA and mtDNA in Q and NQ cells.

### CR increases the resistance of Q and NQ cells to long-term thermal and oxidative stresses

Thermotolerance is one of the characteristic features of Q cells that distinguish them from NQ cells [5, 7]. Moreover, a development of the enhanced resistance to chronic (long-term) thermal and/or oxidative stresses is known to delay aging in yeast and other eukaryotes [1, 2, 4, 103, 155, 159-171].

**Figure 7:**
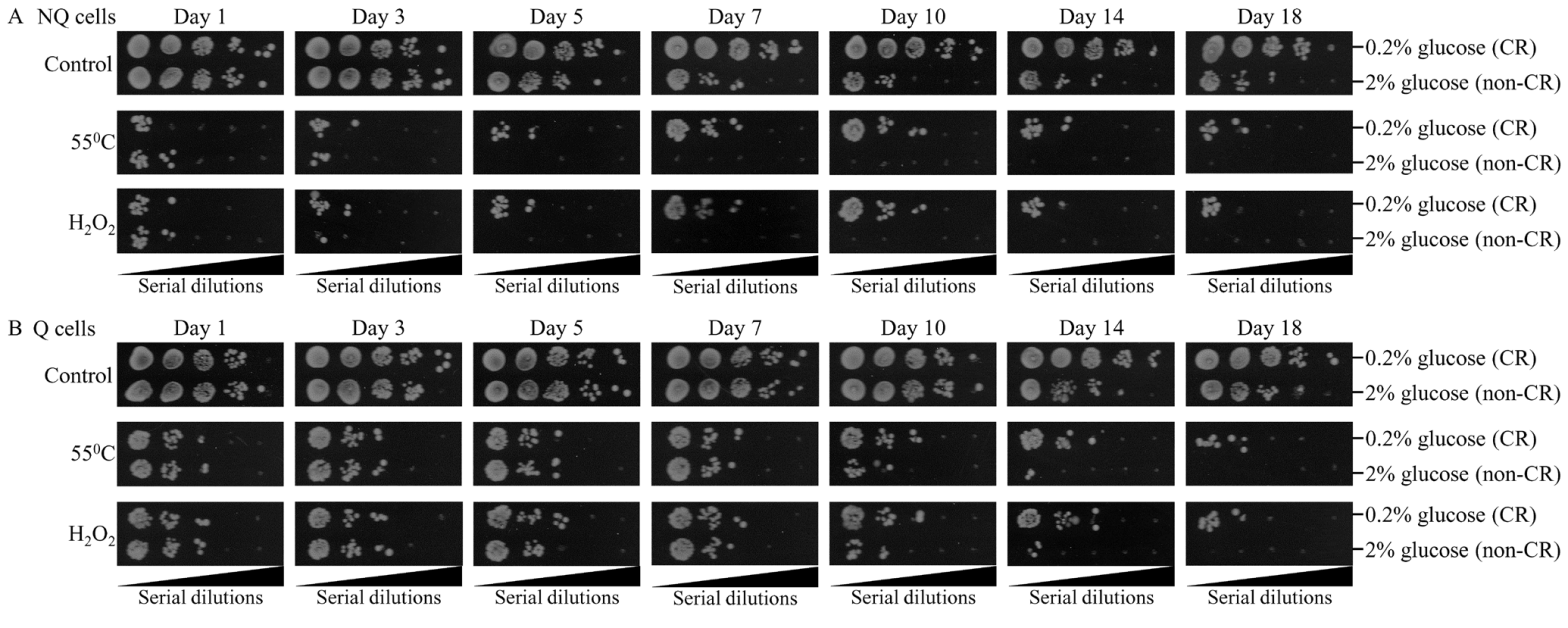
CR increases the resistance of Q and NQ cells to long-term thermal and oxidative stresses Samples of WT yeast cultured in YP medium initially containing 0.2% glucose (CR conditions) or 2% glucose (non-CR conditions) were recovered from L, D, PD or ST growth phase and subjected to centrifugation in Percoll density gradient to purify Q and NQ cell populations, as described in Materials and Methods. Spot assays for monitoring thermal or oxidative stress resistance were performed as described in Materials and Methods. For chronic thermal stress, serial 10-fold dilutions of pure NQ **A.** and Q **B.** cells were spotted on plates with solid YEPD medium containing 2% glucose; these plates were initially exposed to 55°C for 60 min, and then transferred to 30°C and incubated for 3 days. For chronic oxidative stress, serial 10-fold dilutions of pure NQ (A) and Q (B) cells were spotted on plates with solid YEPD medium containing 2% glucose and 5 mM hydroxide peroxide; these plates were then incubated at 30°C for 3 days.

We assessed the effects of CR on the abilities of Q and NQ cell populations to resist chronic oxidative and thermal stresses. Q and NQ cell populations were recovered from differently aged CR or non-CR yeast cultures and purified by centrifugation in Percoll density gradient. Chronic thermal stress was administered by spotting pure Q and NQ cells on plates with solid YEPD medium containing 2% glucose, incubating these plates at 55°C for 60 min, and then transferring them to 30°C and incubating for 3 days. Chronic oxidative stress was applied by spotting pure Q and NQ cells on plates with solid YEPD medium containing 2% glucose and 5 mM hydroxide peroxide, and incubating them at 30°C for 3 days. Our analyses have revealed the following: 1) early in chronological lifespan (i.e. on day 5 for thermal stress or on day 3 for oxidative stress), NQ cells from CR cultures become more resistant to both these kinds of chronic stresses as compared to NQ cells from age-matched non-CR cultures (Figure 7A); and 2) late in chronological lifespan (i.e. since day 10), Q cells from CR cultures exhibit higher resistance to both oxidative and thermal stresses then Q cells from non-CR cultures of the same chronological age (Figure 7B).

Collectively, these findings indicate that CR elicits a significant rise in the resistance of Q and NQ cells to long-term thermal and oxidative stresses. Q cells display the stimulatory effect of CR on the tolerance to both kinds of stresses only late in chronological lifespan, whereas in NQ cells such effect can be seen already early in life.

### CR delays the onsets of the age-related apoptotic and necrotic modes of PCD in Q and NQ populations

In NQ cells from non-CR yeast cultures, the onsets of the age-related apoptotic and necrotic modes of PCD are known to occur much earlier in chronological lifespan than in Q cells from these cultures [5, 7]. Furthermore, chronologically aging yeast cells in non-CR cultures have been shown to die exhibiting characteristic markers of the apoptotic and/or necrotic subroutines of PCD [2, 172-183].

**Figure 8:**
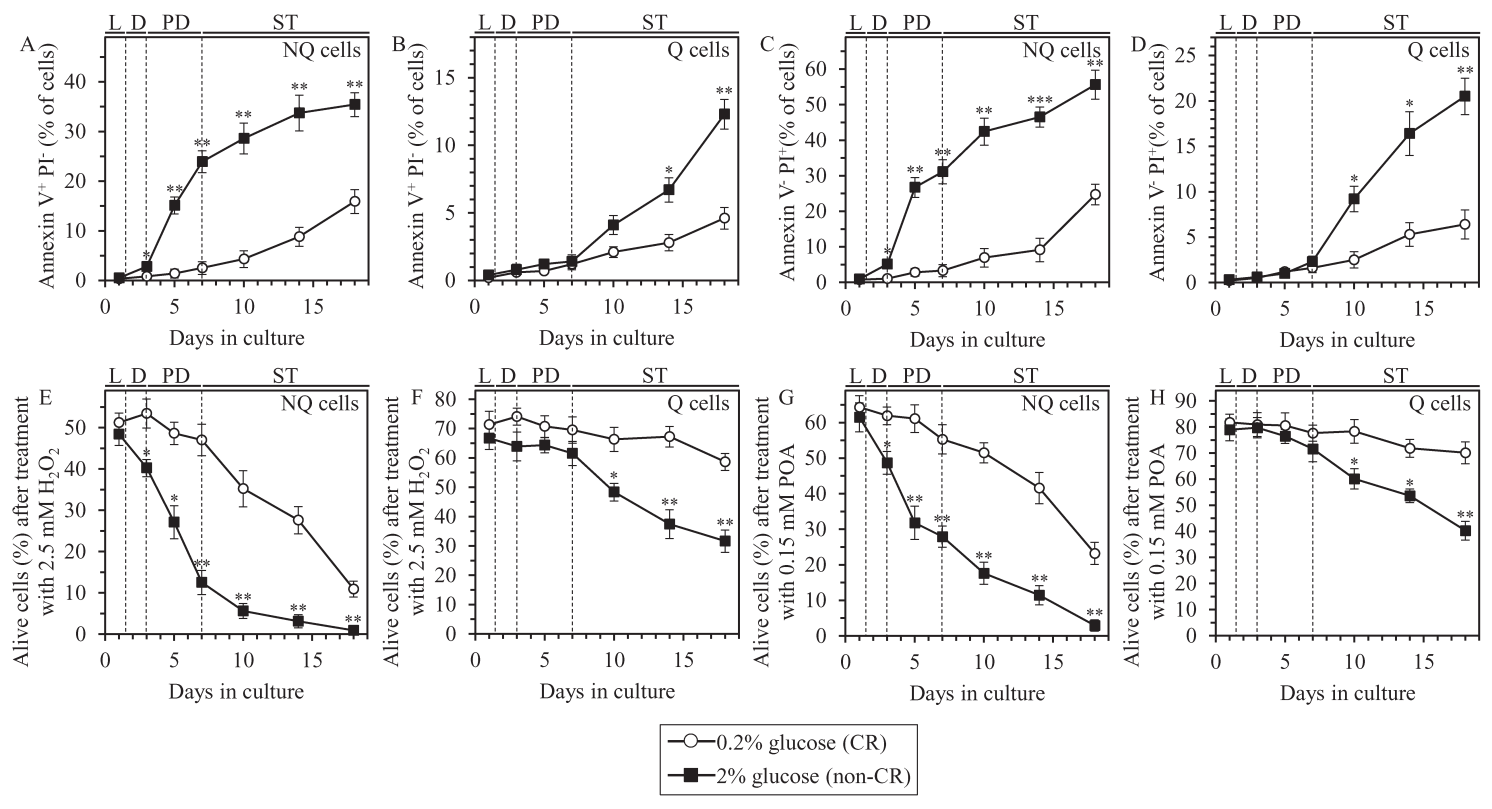
CR causes a decline in the susceptibilities of Q and NQ cells to the exogenously induced apoptotic and liponecrotic modes of PCD Samples of WT yeast cultured in YP medium initially containing 0.2% glucose (CR conditions) or 2% glucose (non-CR conditions) were recovered from L, D, PD or ST growth phase and subjected to centrifugation in Percoll density gradient to purify Q and NQ cell populations, as described in Materials and Methods. Clonogenic assays for monitoring the susceptibilities of NQ (E and G) and Q (F and H) cells to the apoptotic (E and F) or liponecrotic (G and H) mode of PCD induced in response to a short-term (for 2 h) exposure to exogenous 2.5 mM hydrogen peroxide (E and F) or 0.15 mM POA (G and H), respectively, were performed as described in Materials and Methods. Data are presented as means ± SEM (*n* = 3; *P < 0.05; **P < 0.01; ***P < 0.001).

We therefore investigated how CR influences the onsets of age-related the apoptotic and necrotic modes of PCD in Q and NQ cell populations. Q and NQ cell populations were recovered from differently aged CR or non-CR yeast cultures and purified by centrifugation in Percoll density gradient. Apoptotic PCD was microscopically visualized by Annexin V staining for monitoring phosphatidylserine (PS) translocation from the inner to the outer leaflet of the plasma membrane, a characteristic marker of this PCD subroutine. Propidium iodide (PI) staining for measuring the extent of plasma membrane permeability for small molecules, a hallmark of necrotic PCD, was used to microscopically visualize programmed necrosis. Our analyses have revealed the following: 1) through most of the chronological lifespan (i.e. since day 3 of culturing), the percentage of cells undergoing an apoptotic or necrotic mode of PCD in NQ cells from CR cultures is significantly lower than that in NQ cells from age-matched non-CR cultures (Figure 8A and 8C, respectively); and 2) late in chronological lifespan (i.e. since day 14 for apoptotic PCD or day 10 for necrotic PCD), the percentage of cells committed to any of these two PCD modes in Q cells from CR cultures is considerably decreased as compared to that in Q cells from non-CR cultures of the same chronological age (Figures 8B and 8D, respectively).

We therefore concluded that CR decelerates the onsets of age-related apoptotic and necrotic PCD in Q and NQ cell populations. In Q cells these effects of CR take place only late in chronological lifespan, whereas NQ cells exhibit them already early in life.

### CR decreases the susceptibilities of Q and NQ cells to the exogenously induced apoptotic and liponecrotic modes of PCD

Because CR delays the onsets of age-related the apoptotic and necrotic modes of PCD in Q and NQ populations of chronologically aging yeast, we examined how CR influences the susceptibilities of Q and NQ cells to each of these PCD subroutines that were elicited in response to certain exogenous stimuli. A brief exposure of yeast to exogenous hydrogen peroxide is known to decrease clonogenic survival of cells by causing mitochondria-controlled apoptotic PCD [173, 176, 181, 184, 185], whereas a short-term treatment of yeast with exogenous palmitoleic acid (POA) has been shown to reduce clonogenic survival of cells by eliciting a liponecrotic mode of PCD [180-183].

Our comparative analyses have revealed the following: 1) through most of the chronological lifespan (i.e. since day 3 of culturing), clonogenic survival of NQ cells from CR cultures briefly exposed to exogenous hydrogen peroxide (to elicit apoptotic PCD) or POA (to trigger liponecrotic PCD) significantly exceeds that of NQ cells from age-matched non-CR cultures (Figure 8E and 8G, respectively); and 2) late in chronological lifespan (i.e. since day 10), clonogenic survival of Q cells from CR cultures subjected to a short-term treatment with exogenous hydrogen peroxide (to initiate apoptotic PCD) or POA (to induce liponecrotic PCD) is significantly higher than that of Q cells from non-CR cultures of the same chronological age (Figure 8F and 8H, respectively).

In sum, these data indicate that CR causes a decline in the susceptibilities of Q and NQ cell populations to the apoptotic and liponecrotic modes of PCD induced in response to a short-term exposure to exogenous hydrogen peroxide or POA, respectively. In Q cells, the abilities of CR to cause these effects can be seen only late in chronological lifespan, whereas NQ cells display both effects of CR already early in life.

## DISCUSSION

**Figure 9:**
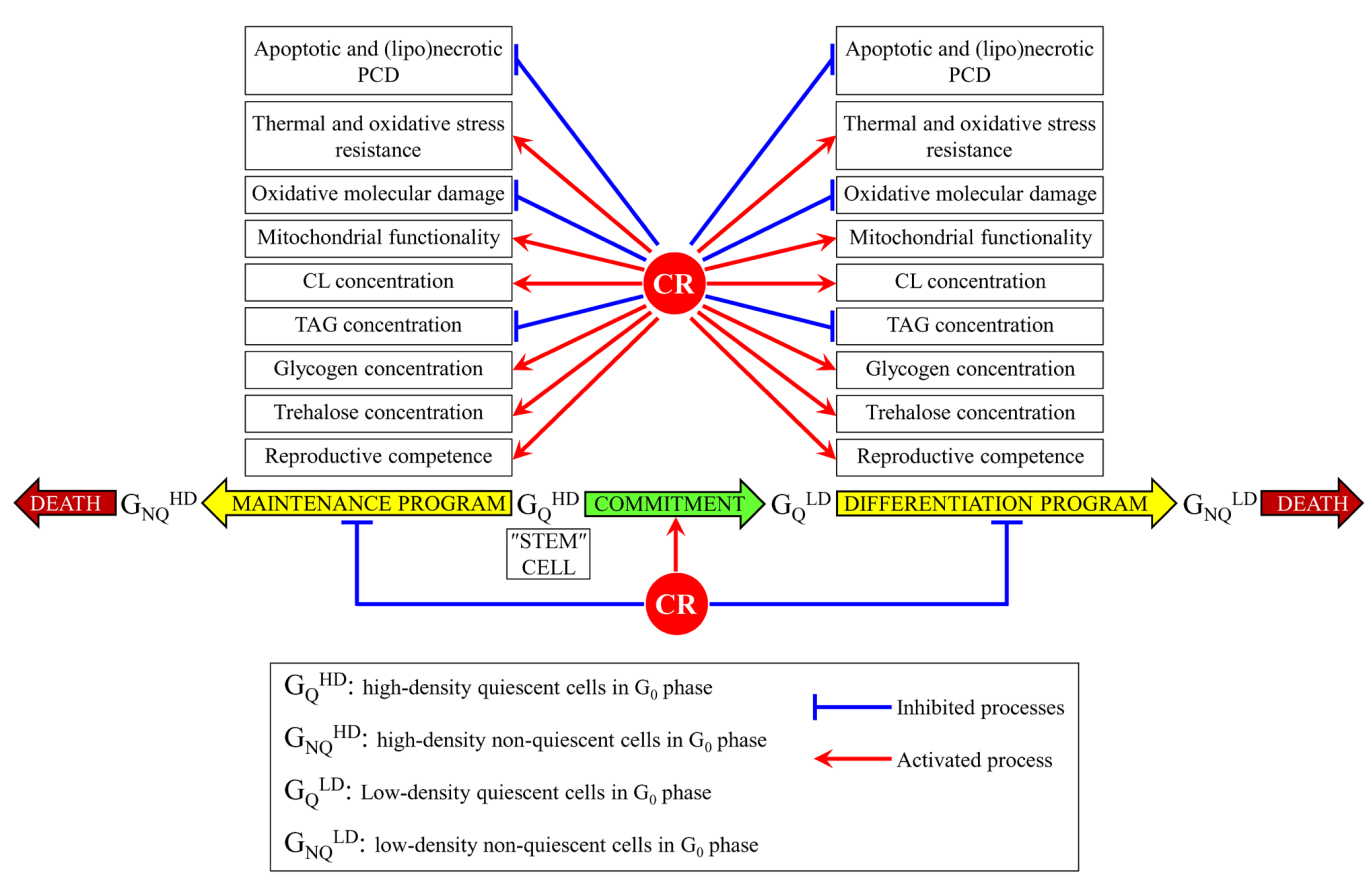
A model for how CR extends yeast chronological lifespan via a mechanism that links cellular aging to cell cycle regulation, maintenance of quiescence, entry into the non-quiescent state and survival in the non-quiescent state CR delays yeast chronological aging by causing specific changes at the G_1_ checkpoint for cell cycle arrest and entry into the G_0_ state, timing of G_Q_^HD^ (high-density, quiescent) cells commitment to conversion into G_Q_^LD^ (low-density, quiescent) cells, pace of progression of G_Q_^LD^ (low-density, quiescent) cells through a differentiation program yielding G_NQ_^LD^ (low-density, non-quiescent) cells, and rate of advancement of GQHD (high-density, quiescent) cells via a maintenance program leading to their conversion into GNQHD (high-density, non-quiescent) cells. Please see text for additional details.

This study revealed that CR extends yeast chronological lifespan via a mechanism that links cellular aging to cell cycle regulation, maintenance of the Q state, entry into the NQ state and survival in the NQ state. Our comparative analyses of physical, morphological, reproductive, biochemical and physiological properties of Q and NQ cells from differently aged CR or non-CR yeast cultures suggest a hypothetical model for this mechanism. This model is depicted schematically in Figure 9.

The model posits that Q and NQ cell populations exist as several subpopulations related to each other in a chronological age-dependent manner. One of these subpopulations is a subpopulation of non-differentiated Q cells that represents a ″stem″ cell niche (Figure 9). We call these stem cells G_Q_^HD^ because they are high-density cells that are arrested at the G_1_ phase of the cell cycle and exist in a specialized non-proliferative state called G_0_. G_Q_^HD^ cells constituting the stem cell niche are viable unbudded cells that 1) exhibit high reproductive (colony-forming) capacity; 2) are able to synchronously re-enter the mitotic cell cycle after cell transfer into fresh medium; 3) display high concentrations of glycogen and trehalose, the two major glucose stores; 4) possess low concentrations of TAG and high concentrations of CL; 5) have fully functional mitochondria; 6) exhibit low concentrations of ROS; 7) display low degree of oxidative damage to proteins, lipids and DNA; 8) are resistant to long-term thermal and oxidative stresses; and 9) display low susceptibilities to the mitochondria-controlled apoptotic and fatty acid-induced liponecrotic forms of PCD.

In L (for CR yeast cultures) or SP (for non-CR yeast cultures) phase, G_Q_^HD^ cells become committed to entry into a program that we call the differentiation program (Figure 9). Such commitment of G_Q_^HD^ cells to differentiation is manifested in a significant decrease of their buoyant density, so that they are converted to a subpopulation of Q cells that we call G_Q_^LD^ cells because of their low density. The chronological age-related progression of G_Q_^LD^ cells through the differentiation program causes a gradual decline in the following features: viability, reproductive (colony-forming) capacity, the ability to synchronously re-enter the mitotic cell cycle after cell transfer into fresh medium, glycogen and trehalose concentrations, TAG concentration, mitochondrial functionality, the ability to maintain low concentration of ROS, the capacity to limit oxidative molecular damage, the tolerance to long-term thermal and oxidative stresses, and the resistance to the apoptotic and liponecrotic modes of PCD. The progression of G_Q_^LD^ cells through the differentiation program ultimately leads to their conversion into a subpopulation of NQ cells that we call G_NQ_^LD^ cells; these cells are committed to the programmed, age-related modes of apoptotic and/or liponecrotic cell death (Figure 9).

When a yeast culture enters ST phase, most (for CR yeast cultures) or many (for non-CR yeast cultures) of non-differentiated G_Q_^HD^ cells from the stem cell niche have been committed to entry into and progression through the differentiation program and, thus, exist in the G_Q_^LD^ and G_NQ_^LD^ forms. However, a portion of cells within this culture still remains in the G_Q_^HD^ form (Figure 9). Through the entire chronological lifespan, these non-differentiated stem cells progress through a program which we call the maintenance program. A progression of G_Q_^LD^ stem cells through this maintenance program is manifested in a much slower deterioration in the same features as the ones whose relatively fast decline occurs during the differentiation program (Figure 9). The advancement of G_Q_^HD^ cells through the maintenance program eventually results in their conversion into a subpopulation of NQ cells that we call G_NQ_^HD^ cells; these cells are susceptible to the chronological age-related subroutines of apoptotic and/or liponecrotic PCD (Figure 9).

Our findings suggest that CR delays yeast chronological aging by causing specific changes in a G_1_ checkpoint for cell cycle arrest and entry into the G_0_ state, a growth phase in which G_Q_^HD^ cells undergo the commitment to become G_Q_^LD^ cells, the differentiation of G_Q_^LD^ cells into G_NQ_^LD^ cells, and the conversion of G_Q_^HD^ cells into G_NQ_^HD^ cells. These changes are described below.

Judging from the very small size of G_Q_^HD^ stem cells seen in CR cultures (Figure 1G), CR may arrest the cell cycle at a previously unknown checkpoint in early G_1_. This is unlike the large size of G_Q_^HD^ stem cells observed in non-CR cultures (Figure 1G), whose cell cycle is known to be arrested at the checkpoint START A in late G_1_ [14, 186].

Furthermore, CR is likely to accelerate the onset of commitment of G_Q_^HD^ stem cells to entry into the differentiation program (Figure 9). Indeed, G_Q_^HD^ cells in CR cultures become committed to entry into this program already in L growth phase, whereas in CR cultures such commitment occurs only in SP phase (Figure 1D).

Moreover, our data indicate that CR can also decrease both the pace of progression of G_Q_^LD^ cells through the differentiation program and the rate of advancement of G_Q_^HD^ cells via the maintenance program (Figure 9). These effects of CR slow down yeast chronological aging because they delay the conversion of these two subpopulations of Q cells into G_NQ_^LD^ and G_NQ_^HD^ subpopulations of NQ cells (respectively), both of which are committed to the chronological age-related modes of apoptotic and liponecrotic PCD (Figure 9). Our findings suggest that the observed abilities of CR to decelerate the differentiation and maintenance programs are due to specific effects of this low-calorie diet on a distinct set of morphological, reproductive, biochemical and physiological processes in G_Q_^LD^ and G_Q_^HD^ cells (as outlined above, all these processes have been implicated in defining longevity of chronologically aging yeast). These specific effects of CR in G_Q_^LD^ and G_Q_^HD^ cells include the following: 1) CR slows a chronological age-related deterioration in the reproductive competences of these cells and in their capabilities to synchronously re-enter the mitotic cell cycle when nutrients become available; 2) CR increases glycogen and trehalose concentrations in these cells; 3) CR decreases the concentrations of TAG and raises CL concentrations in these cells; 4) CR improves mitochondrial functionality in these cells; 5) CR reduces ROS concentrations and lessens the degree of oxidative molecular damage in these cells; 6) CR increases the resistance of these cells to long-term thermal and oxidative stresses; and 7) CR decreases the susceptibilities of these cells to the apoptotic and liponecrotic forms of PCD (Figure 9).

The challenge for the future is to test our hypothesis that CR decelerates the differentiation and maintenance programs of G_Q_^LD^ and G_Q_^HD^ cells (respectively) because this low-calorie diet modulates the above-mentioned set of longevity-defining processes. To address this challenge, it would be important to assess how genetic interventions impairing each of these processes may influence the chronological age-related relative abundance of G_Q_^HD^, G_Q_^LD^, G_NQ_^HD^ and G_NQ_^LD^ subpopulations in CR and non-CR yeast cultures. It would be also interesting to examine how these genetic interventions may affect longevity of chronologically aging yeast under CR and non-CR conditions.

Another challenge for the future is to explore how genetic and pharmacological interventions known to delay yeast chronological aging by modulating a network of certain signaling pathways and protein kinases may impinge on the described here mechanism that links cellular aging to cell cycle regulation, maintenance of the Q state, entry into the NQ state and survival in the NQ state. This network integrates the TORC1, PKA, Snf1 and Pho85 core hubs of the signaling network of quiescence, as well as their downstream effector proteins Sch9, Rim15 and others [1, 12, 14, 21, 28, 144, 175, 187-192].

## MATERIALS AND METHODS

### Yeast strains, media and growth conditions

The wild-type strain *Saccharomyces cerevisiae* BY4742 (*MAT*a *his3D1 leu2D0 lys2D0 ura3D0*) from Open Biosystems/Dharmacon (a part of GE Healthcare) was grown in YP medium (1% yeast extract, 2% peptone; both from Fisher Scientific; #BP1422-2 and #BP1420-2, respectively) initially containing 0.2% or 2% glucose (#D16-10; Fisher Scientific) as carbon source. Cells were cultured at 30^°^C with rotational shaking at 200 rpm in Erlenmeyer flasks at a ″flask volume/medium volume″ ratio of 5:1.

### Separation of quiescent and non-quiescent cells by centrifugation in Percoll density gradient

1 ml of 1.5 M NaCl (#S7653; Sigma) was placed into a 50-ml conical polypropylene centrifuge tube (#055398; Fisher Scientific), and 8 ml of the Percoll solution (#P1644; Sigma) was added to this tube. The NaCl and Percoll solutions were then mixed by pipetting. To form two Percoll density gradients, 4 ml of the NaCl/Percoll mixture was put into each of the two polyallomer tubes for an MLS-50 rotor for an Optima MAX ultracentrifuge (all from Beckman Coulter, Inc.). The tubes were centrifuged at 25,000 × g (16,000 rpm) for 15 min at 4°C in an Optima MAX ultracentrifuge. A sample of yeast cells was taken from a culture at a certain time-point. A fraction of the sample was diluted in order to determine the total number of cells per ml of culture using a hemacytometer (#0267110; Fisher Scientific). For each Percoll density gradient, 1 × 10^9^ yeast cells were placed into a 15-ml conical polypropylene centrifuge tube (#0553912; Fisher Scientific) and then pelleted by centrifugation at 5,000 rpm for 7 min at room temperature in an IEC Centra CL2 clinical centrifuge (Thermo Electron Corporation). Pelleted cells were resuspended in 500 μl of 50 mM Tris/HCl buffer (pH 7.5), overlaid onto the preformed Percoll gradient and centrifuged at 2,300 × g (5,000 rpm) for 30 min at 25°C in an Optima MAX ultracentrifuge. The upper and lower fractions of cells were collected with a pipette, Percoll was removed by washing cells twice with 50 mM Tris/HCl buffer (pH 7.5) and cells were resuspended in 50 mM Tris/HCl buffer (pH 7.5) for subsequent assays.

### Reproductive (colony forming) capability assay for quiescent and non-quiescent cells separated by centrifugation in Percoll density gradient

An aliquot of the upper or lower fraction of cells recovered from the Percoll gradient and washed twice with 50 mM Tris/HCl buffer (pH 7.5) was diluted in order to determine the total number of cells per fraction using a hemacytometer (#0267110; Fisher Scientific). Serial dilutions (1:10^2^ to 1:10^5^) of cells were also plated onto YEPD (1% yeast extract, 2% peptone, 2% glucose) plates in duplicate in order to count the number of viable cells per ml of each cell fraction. 100 ml of diluted culture was plated onto each plate. After 48-h incubation at 30°C, the number of colonies per plate was counted. The number of colony forming units (CFU) equals to the number of reproductively capable cells in a sample. Therefore, the number of reproductively capable cells was calculated as follows: number of CFU × dilution factor × 10 = number of reproductively capable cells per ml. For each cell fraction assayed, % reproductive capability of the cells was calculated as follows: number of CFU per ml/total number of cells per ml × 100%.

### Synchronous reentry into mitosis assay for quiescent and non-quiescent cells separated by centrifugation in Percoll density gradient

5 × 10^6^ cells recovered in the upper or lower fraction of the Percoll gradient and washed twice with 50 mM Tris/HCl buffer (pH 7.5) were harvested by centrifugation for 1 min at 21,000 × g at room temperature. Pelleted cells were washed twice with water and then inoculated into 50 ml of YP medium (1% yeast extract, 2% peptone; both from Fisher Scientific; #BP1422-2 and #BP1420-2, respectively) initially containing 0.2% or 2% glucose (#D16-10; Fisher Scientific) as carbon source. Cells were cultured for 4 h at 30°C with rotational shaking at 200 rpm in Erlenmeyer flasks at a “flask volume/medium volume” ratio of 5:1. A sample of cells was taken from a culture at a certain time-point and examined microscopically for the percentage of cells with new buds. At least 500 cells were examined per time point, and the budding percentage was calculated as follows: (number of cells with new buds per ml/total number of cells per ml) × 100%.

### Miscellaneous procedures

Chronological lifespan analysis [4], a microanalytic biochemical assay for measuring glucose concentration [193], cell size and number measurements [4], a Calcofluor White M2R (#L7009, component B; Molecular Probes/Thermo Fisher Scientific) staining for microscopical visualization of bud scars [8], a measurement of the number of bud scars per cell [8], glycogen and trehalose measurements [194], a mass spectrometric quantitative assessment of the yeast lipidome [194], cellular respiration measurement [4], fluorescence microscopy for measuring ROS and ΔΨ_m_ [4], a measurement of the frequency of spontaneous point mutations in the *CAN1* gene of nDNA by monitoring the frequency of mutations that caused resistance to the antibiotic canavanine (#C1625; Sigma) [4], a measurement of the frequency of spontaneous point mutations in the *rib2* and *rib3* loci of mtDNA by monitoring the frequency of mtDNA mutations that caused resistance to the antibiotic erythromycin (#227330050; Acros Organics) [4], oxidatively damaged proteins and lipid measurements with a Protein Carbonyl Assay Kit assay kit (#10005020; Cayman Chemical) and a PeroXOquant Quantitative Peroxide Assay Kit assay kit (#23285; Thermo Scientific Pierce), respectively [181], a plating assay for analyzing the resistance to thermal and oxidative stresses [195], an Annexin V (#630109; Clontech Laboratories, Inc.) staining for microscopical visualization of externalized PS and a PI (#P3566; Life Technologies/Molecular Probes) staining for microscopical visualization of the extent of plasma membrane permeability for small molecules [4], a cell viability assay for monitoring the susceptibility of yeast to an apoptotic mode of cell death induced by hydrogen peroxide [181], and a cell viability assay for monitoring the susceptibility of yeast to a liponecrotic mode of cell death induced by POA [181] were performed as previously described.

### Statistical analysis

Statistical analysis was performed using Microsoft Excel’s (2010) Analysis ToolPak - VBA. All data on cell survival are presented as mean ± SEM. The P values for comparing the means of two groups using an unpaired two-tailed *t* test were calculated with the help of the GraphPad Prism 7 statistics software. The logrank test for comparing each pair of survival curves was performed with GraphPad Prism 7. Two survival curves were considered statistically different if the P value was less than 0.05.

## SUPPLEMENTARY MATERIALS FIGURE



## Abbreviations

CDK: cyclin-dependent kinase
CFU: colony forming units
CL: cardiolipins
CR: caloric restriction
D: diauxic growth phase
HD: high-density cell population
L: logarithmic growth phase
LD: low-density cell population
mtDNA: mitochondrial DNA
NQ: non-quiescent cells
PCD: programmed cell death
PD: post-diauxic growth phase
POA: palmitoleic acid
Pho85: phosphate metabolism, protein 85
PS: phosphatidylserine
PI: propidium iodide
PKA: protein kinase A; Q, quiescent cells
ROS: reactive oxygen species
SNF: sucrose non-fermenting, protein 1
ST: stationary growth phase
TAG: triacylglycerols
TORC1: target of rapamycin complex 1
YEPD: 1% yeast extract + 2% peptone + 2% glucose; YP, 1% yeast extract + 2% peptone
ΔΨ_m_: mitochondrial membrane potential
