# Supplementary material for: Caloric restriction extends yeast chronological lifespan via a mechanism linking cellular aging to cell cycle regulation, maintenance of a quiescent state, entry into a non-quiescent state and survival in the non-quiescent state
Source: Oncotarget. 2017 Sep 1;8(41):69328–50. doi: 10.18632/oncotarget.20614 (PMC5642482; doi:10.18632/oncotarget.20614)
Supplement: Supplementary file 1 [file oncotarget-08-69328-s001.pdf]

Caloric restriction extends yeast chronological lifespan via a mechanism linking cellular aging to cell cycle regulation, maintenance of a quiescent state, entry into a non-quiescent state and survival in the non-quiescent state

SUPPLEMENTARY MATERIALS

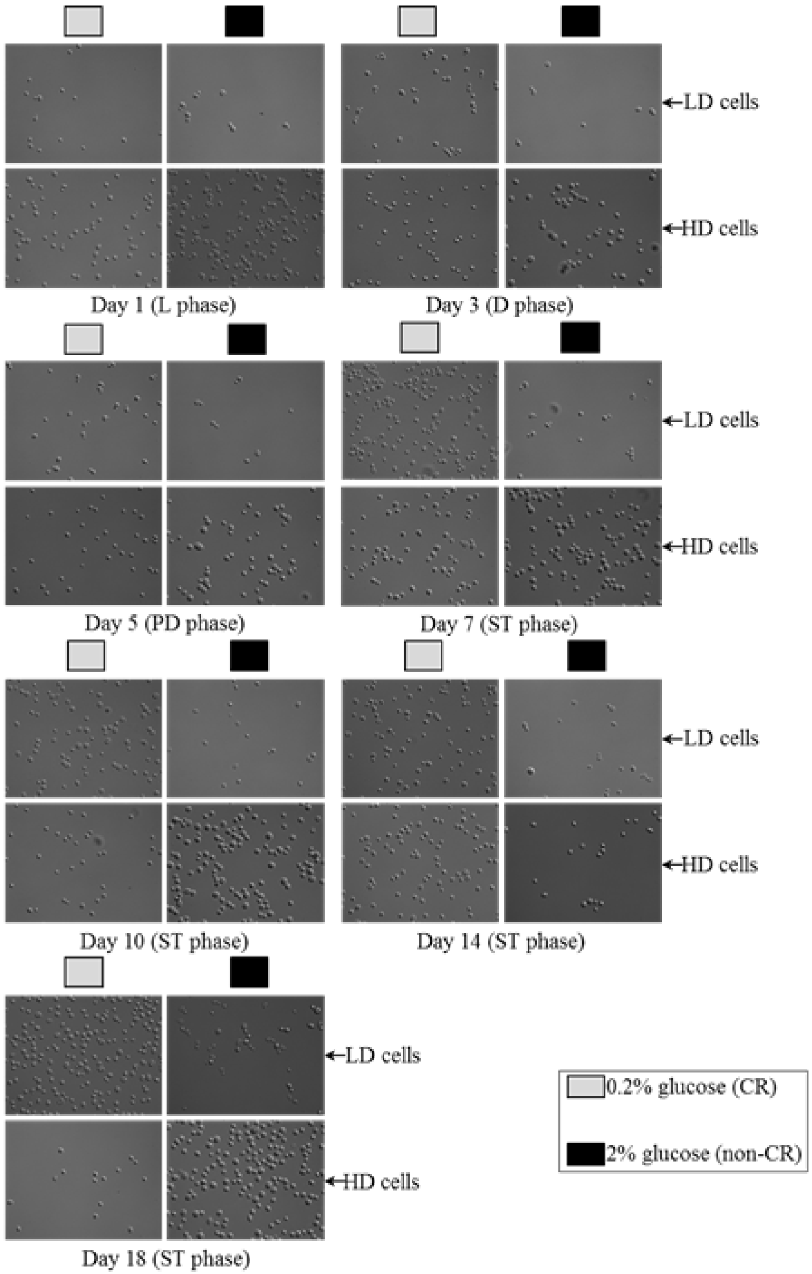

Supplemental Figure 1: Differential interference contrast micrographs of purified LD and HD cells. Samples of wild-type yeast cultured in YP medium initially containing 0.2% glucose (CR conditions) or 2% glucose (non-CR conditions) were recovered from logarithmic (L), diauxic (D), post-diauxic (PD) or stationary (ST) growth phase and subjected to centrifugation in Percoll density gradient to purify LD and HD cell populations.

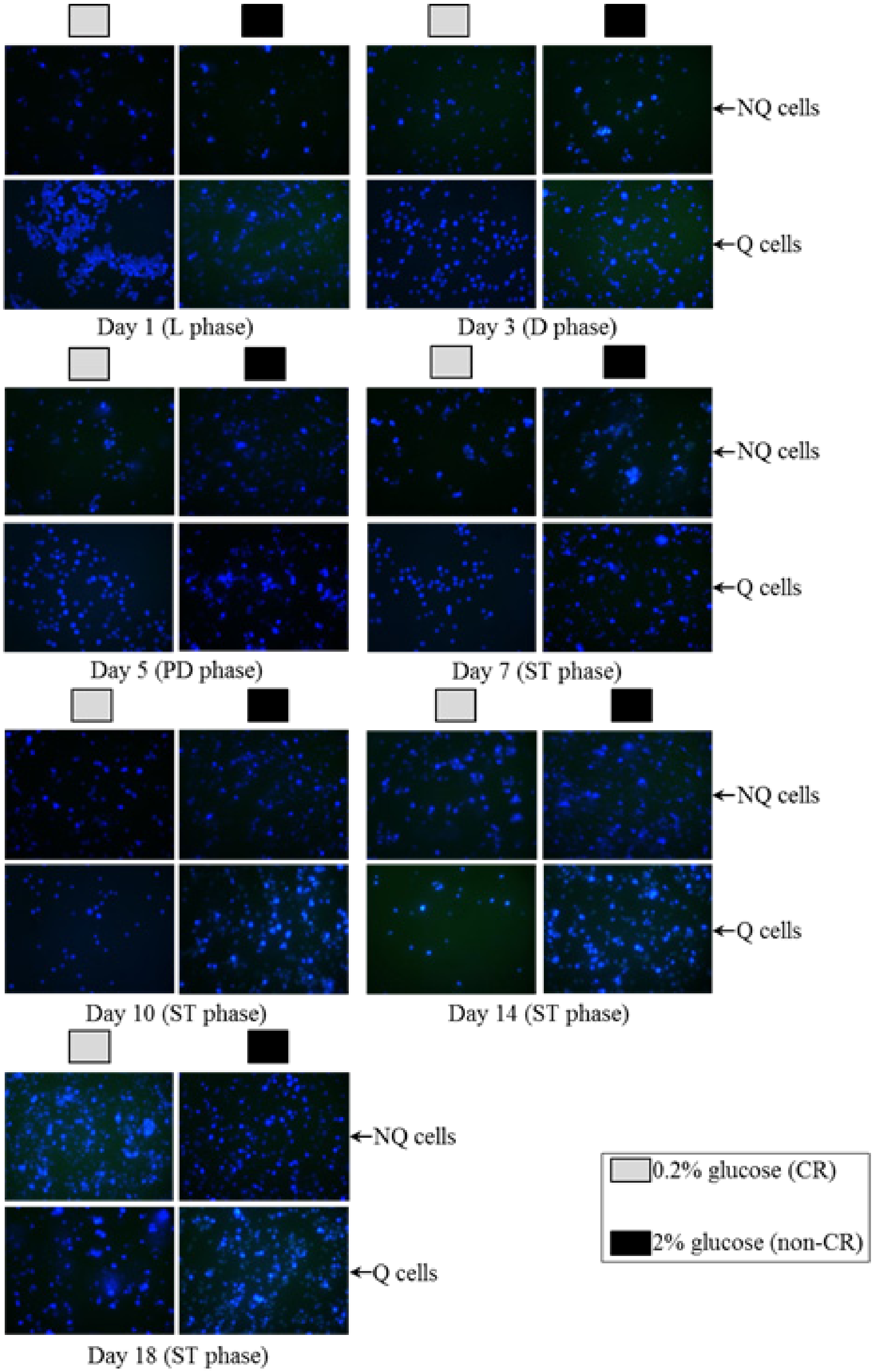

Supplemental Figure 2: CR rises the fraction of daughter cells in Q and NQ populations and, late in chronological lifespan, prevents budding of daughter cells present in both populations. Samples of WT yeast cultured in YP medium initially containing 0.2% glucose (CR conditions) or 2% glucose (non-CR conditions) were recovered from L, D, PD or ST growth phase and subjected to centrifugation in Percoll density gradient to purify Q and NQ cell populations, as described in Materials and Methods. Bud scars were microscopically visualized by staining with Calcofluor White M2R, as described in Materials and Methods.
